# A protocol for the generation of EPO - Producing neural crest cells from human induced pluripotent stem cells

**DOI:** 10.1016/j.mex.2022.101753

**Published:** 2022-06-09

**Authors:** Angelo Michele Lavecchia, Sara Buttò, Christodoulos Xinaris

**Affiliations:** Istituto di Ricerche Farmacologiche Mario Negri IRCCS, Bergamo, Italy

**Keywords:** Erythropoietin, Human induced pluripotent stem cells, Neural crest cells, Anaemia

## Abstract

Insufficient production of erythropoietin (EPO) leads to anaemia. Developing methods for the generation and transplantation of EPO-producing cells would allow scientists to design personalised therapeutic solutions. Here we present a simple and highly reproducible protocol for the generation of neural crest cells (NCCs) that can produce and secrete erythropoiesis-competent EPO in response to hypoxia.

## Graphical Abstract

**Figure:**
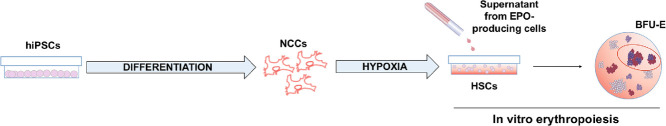

Specifications table

**Table:** 

Subject Area:	Biochemistry, Genetics and Molecular Biology
More specific subject area:	Erythropoietin – producing neural crest cells
Protocol name:	Differentiation of erythropoietin – producing neural crest cells from human induced pluripotent stem cells
Reagents/tools:	Included in each section of the protocol
Experimental design:	Human induced pluripotent stem cells are cultured in a chemically defined medium to differentiate into neural crest cells. Differentiated cells can produce functional erythropoietin in response to hypoxia.
Trial registration:	N/A
Ethics:	N/A
Value of the Protocol:	• This is a simple and reproducible differentiation protocol to generate neural crest cells from hiPSCs • The differentiated neural crest cells are fully competent to produce functional erythropoietin in response to hypoxia • This protocol can provide the basis for the development of a novel therapeutic cell-based approach to treat anaemia

## Description of protocol

In patients who suffer from chronic kidney diseases (CKD), the renal erythropoietin (EPO)-producing (REP) cells, which release EPO under hypoxic conditions and stimulate erythropoiesis, lose their capacity to produce EPO, leading to renal anaemia. The main available treatment for these patients is recombinant human erythropoietin (rhEPO) administration, which remains subject to various limitations, such as its high cost and adverse effects.

**Fig. 1:**
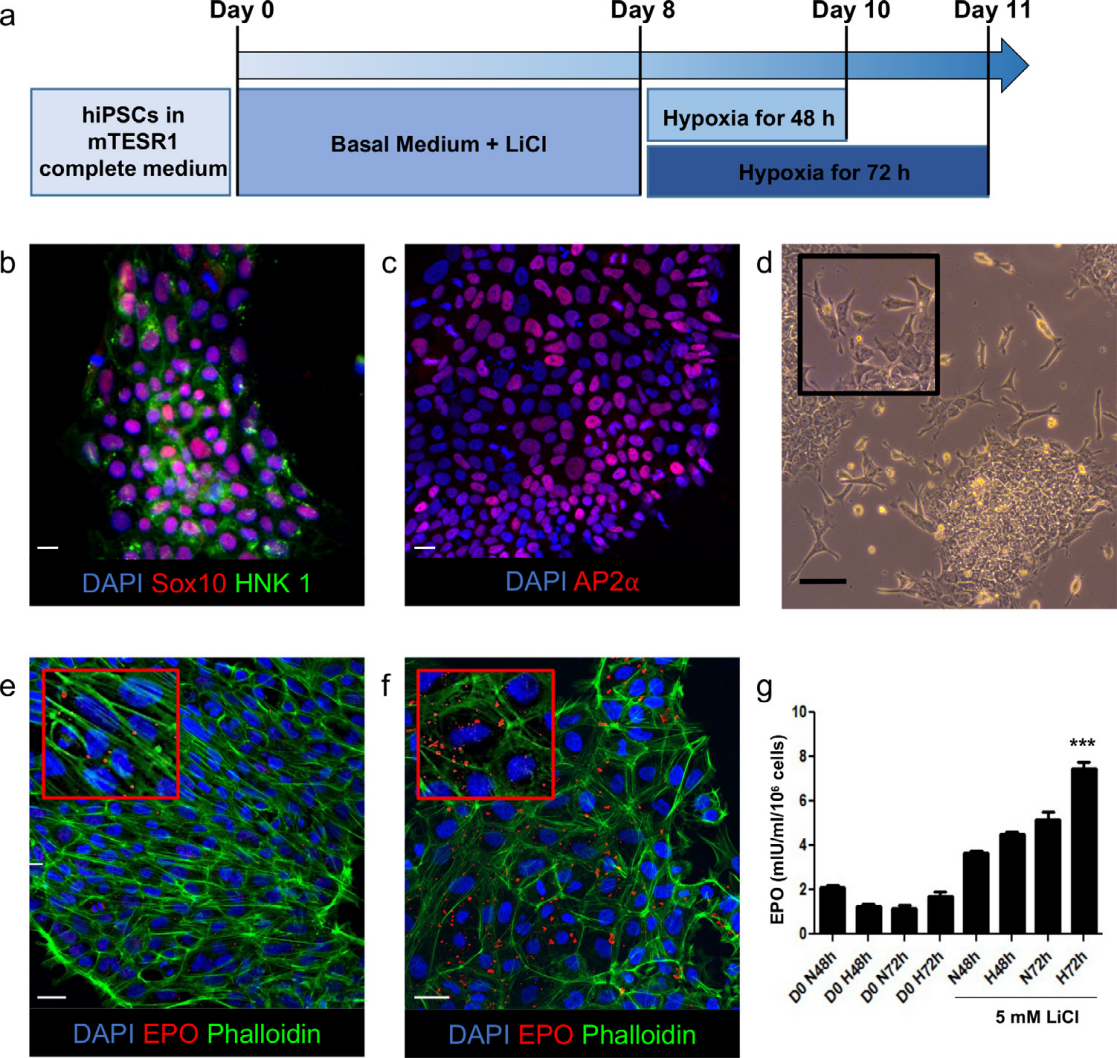
(a) Schematic representation of the differentiation protocol. Exposure to Basal Medium supplemented with 5 mM of fresh lithium chloride for 8 days induced hiPSCs to differentiate into NCCs. Neural crest cells were then exposed to normoxic or hypoxic conditions for 48 and 72 hours to evaluate their capacity to secrete functional EPO. (b, c) Immunostaining analysis for neural crest cells specific markers (b) Sox10, HNK1 and (c) AP2⍺ at day 8 of differentiation. (d) Bright field microscopy image of migrating cells at day 7 of differentiation. (e, f) Immunofluorescent images of EPO production in (e) normoxic and (f) hypoxic conditions. EPO is secreted in cell cytoplasm (inset). (g) EPO concentration in the supernatant of undifferentiated hiPSCs (d0, control) and NCCs under normoxic or hypoxic conditions for 48h and 72h. ***p<0.001 vs all culture conditions tested by one - way ANOVA. N, normoxia; H, hypoxia; h, hours. Digital images (b, c, e, f) were acquired using an inverted confocal laser microscope (Leica TCS SP8, Leica Biosystems) or (d) Apotome Axio Imager Z2 (Carl Zeiss). Scale bars (b, c) 10 μm, (d) 100 μm, (e, f) 50 μm. More information about NCCs’ characterization and immonofluorescence protocols can be found in the original paper.

Here we report a protocol to differentiate human induced pluripotent stem cells (hiPSCs) into neural crest cells (NCCs) and show that these cells can produce functional EPO when cultured under hypoxic conditions (Fig. 1a), and induce erythropoiesis both in vitro and in vivo.

## Method details

### Differentiation of hiPSCs into Neural Crest Cells

#### Materials and reagents

- Human Induced Pluripotent Stem Cells (hiPSCs) (clone IV; RRID:CVCL IT61) [1,2];
- 100 × 20 mm Petri Dishes, Nunclon^TM^ Delta (Cat#353003, Corning, NY 14831 USA);
- 6 multiwells cell culture plate (Cat#CLS3516 Corning, NY 14831 USA);
- Stem Pro Accutase (Cat#A1110501; ThermoFisher), 4°C;
- Growth-Factor-Reduced Basement Membrane Matrix, phenol red-free (Cat#356231, Corning, NY 14831 USA) -20°C. Thaw Matrigel® overnight in a refrigerator (4°C) by submerging the vial in a bucket filled with ice, aliquot and store at – 80°C until use. Thaw, on ice, the appropriate number of aliquots immediately before use;
- mTeSR1 medium (Cat#05850, StemCell Technologies, Vancouver, Canada) enriched with mTeSR1 5x Supplement;
- Lonza^TM^ BioWhittaker^TM^ Phosphate Buffered Solution (PBS) (1X) (Cat#BE17-516F, Lonza), without Ca^2+^ and Mg^2+^, room temperature;
- Y27632 dihydrochloride [Rho-associated protein (ROCK) inhibitor] 10 μM (Cat#Y0503, Sigma), aliquot and store at – 80°C;
- 1000 μL adjustable single channel micropipette (P1000) with disposable sterile tips.

Basal Medium for differentiation to Neural Crest Cells:

- DMEM/F12+GlutaMAX^TM^ Supplemented (Cat#31331, ThermoFisher), 4°C;
- CellMaxx Bovine Albumin 17.5 mg/ml (Cat#0219989925, MP BiomedicalsTM), 4°C;
- Human insulin 17.5 μg/ml (Cat#I9278, Sigma), 4°C;
- Human holo-transferrin 275 μg/ml (Cat#T0665, Sigma), 4°C;
- 1-thioglycerol 450 μM (Cat#M6145, Sigma), 4°C;
- Non-essential amino acids 0.1 mM (Cat#11140050, ThermoFisher), 4°C;
- Lithium chloride 5 mM (LiCl; L0505, Sigma), room temperature;

Human iPSCs clone IV was obtained from neonatal fibroblasts using STEMCCA lentivirus. Cells were maintained in mTeSR1 complete medium and cultured under standard conditions (37°C, 5% CO_2_, 20% O_2_) on Growth-Factor-Reduced Basement Membrane Matrix-coated 100 × 20 mm Petri Dishes. Medium was changed daily. When 80% confluent, hiPSCs can be harvested as follows:

1. Wash the cells twice with prewarmed PBS 1X.
2. Remove PBS 1X, add 3 mL Accutase and incubate the plate for 3 minutes at 37°C to detach the cells.
3. Collect the cells by gently pipetting with a P1000, count them, and make an aliquot of 3 × 10^4^ viable cells.
4. Centrifuge at 200 x g for 5 minutes.
5. Resuspend in mTeSR1 complete medium containing 10 μM ROCK-inhibitor (2 mL/well) and seed with a concentration of 3 × 10^4^ viable cells/cm^2^ onto Growth-Factor-Reduced Basement Membrane Matrix-coated 6-well cell culture plate for 24 hours.
6. After 24 hours, remove the medium from each well and replace it with the same amount of Basal Medium supplemented with 5 mM of fresh lithium chloride for 8 days. Change the medium daily.
7. By day 8 of differentiation, the majority of differentiated cells express NCC-specific markers such as AP2α, SOX10 and HNK1 (Fig. 1b, c). For full differentiation markers analysis see the original paper [3].

Note: 24 hours after medium change, cells are organised into small groups interconnected by cellular ramification. At day 2 of differentiation there are clusters of various sizes characterised by more flattened and elongated cells on the edges. Between days 3 and 4 cells undergo a significant amount of cell death. The first migrating cells can be observed at day 6 or 7 of differentiation (Fig. 1d). During differentiation, cells change morphology by replacing the keratin filaments with vimentin intermediate filaments at the level of lamellipodial regions.

### In vitro production of functional EPO

#### Materials and Reagents

- Incubator for Hypoxia (5% O_2_, 37°C, 5% CO_2_)
- Trypsin-EDTA 0.5%, no phenol red (Cat#15400054, Thermofisher Scientific), 4°C. Before use, dilute 1:10 using a balanced salt solution without calcium and magnesium. Aliquots can be stored at – 20°C until use.
- 1000 μL adjustable single channel micropipette (P1000) with disposable sterile tips.
- Sterile safe-lock 1.5 mL microcentrifuge tubes (Cat# T9661-1000EA; Eppendorf®).
- LonzaTM BioWhittakerTM Phosphate Buffered Solution (PBS) (1X) (Cat#BE17- 516F, Lonza), without Ca2+ and Mg2+, room temperature
- Paraformaldehyde (PFA) 8% aqueous solution (Cat#157-8, Electron Microscopy Sciences), room temperature, made up to 4% in PBS 2X. Aliquoted and stored at – 20°C until use.
- Human EPO ELISA kit (Cat#ab119522, Abcam), 4°C
  1. At day 8 of differentiation, change the medium for the last time by adding 2 mL/well of fresh complete Basal Medium supplemented with lithium chloride.
  2. Transfer the plates into the incubator for hypoxia for 48 or 72 hours. Do not change the medium during this period of time.

At the end of hypoxia, remove the plates from the incubator and prepare the sample for EPO functional assay, ELISA, real time qRT-PCR and immunofluorescence analysis:

1. For the functional assay and ELISA test, collect and pool together the supernatant from two wells at a time in a microcentrifuge tube. Measure the total volume of the collected supernatant, aliquot and store at - 20°C until use.
2. Wash the cells twice with PBS 1X, detach them by adding 0.5 mL of prewarmed Trypsin-EDTA 0.05% to each well and incubate for 2 minutes at 37°C. Count the cells from two wells at a time in order to relate the cell number to the EPO production for ELISA analysis.
3. For immunofluorescence analysis, wash the cells twice with PBS 1X and fix them in 4% PFA for 10 minutes at room temperature. Once fixed, they can be preserved in PBS 1X at 4°C for extended periods (several months).
4. For real time qRT-PCR detach the cells by adding 0.5 mL of prewarmed Trypsin-EDTA 0.05% in each well and incubate for 2 minutes at 37°C. Collect the cells, count them and centrifuge 200 x g for 5 minutes. Store the cell pellet at – 80°C.

Immunofluorescence analysis of differentiated NCCs showed that there was an increased EPO production in hypoxic conditions compared to normoxia (Fig. 1e, f). This observation was further confirmed by ELISA analysis of EPO released in NCCs supernatant (Fig. 1g).

To evaluate if the EPO produced was fully functional, we tested its capacity to induce in vitro differentiation of CD34^+^ haematopoietic stem cells (HSCs) into erythroblasts by following a clonogenic (colony formation) assay [3,4]. The group exposed to the EPO-containing supernatant formed significantly more erythroid colonies than the control group, confirming NCC-produced EPO functionality. Moreover, hiPSC-NCCs can be transplanted directly under the skin of anaemic mice to treat anaemia. Cell transplantation was shown to accelerate haematocrit restoration and to induce splenic erythropoiesis after anaemia. The detailed protocols of immunofluorence analysis and functional studies can be found in the original article [3].

## Funding

AML is the recipient of a fellowship from Fondazione Aiuti per la Ricerca sulle Malattie Rare, Bergamo, Italy. AMLand CX’s research is funded by Euronanomed (an ERA-NET grant, EURONANOMED 2019-049/REASON), Associazione per la Ricerca sul Diabete Italia (ARDI), Fondazione Terzi-Albini.

## Declaration of Competing Interests

The authors declare that they have no known competing financial interests or personal relationships that could have influenced the work reported in this paper.
